# The relationship between teacher care behavior and EFL learning anxiety: the chain mediation effect of learning engagement and learning strategies

**DOI:** 10.3389/fpsyg.2023.1279025

**Published:** 2023-11-03

**Authors:** Dongmei Wang

**Affiliations:** School of Foreign Languages, Hubei University of Economics, Wuhan, China

**Keywords:** teacher care behavior, EFL learning anxiety, learning engagement, English learning strategy, mediation effect

## Abstract

This study aims to discuss the impact of teacher care behavior on EFL learning anxiety, as well as the mediating role of learning engagement and learning strategies. The Process plugin Model6 was used to measure the interaction between teacher care behavior (TCB), EFL learning anxiety (EFLLA), learning engagement (LE), and English learning strategies (ELS), in order to analyze and summarize their impact characteristics on college students’ EFL learning anxiety. The results show that teacher care behavior has a direct impact on EFL learning anxiety and a significant negative predictive effect on it. Learning engagement and English learning strategies play a mediating and chain mediating role between teacher care behavior and EFL learning anxiety. Thus, it can be seen that these factors can help reduce EFL learning anxiety, manifested in aspects such as mood, cognition, emotion, and behavior. Given the research findings, this study further provides suggestions for alleviating college students’ EFL learning anxiety, optimizing English teaching and learning design, and cultivating English learning strategies for college students.

## Introduction

In China, English is the most important foreign language with a majority of people learning English as foreign language. Thus, people tend to use foreign language to refer to English (in this study, foreign language classroom means English classroom). In most parts of China, English has always been the core curriculum of primary and secondary schools, and college English is a compulsory course. Even so, most Chinese students still experience anxiety in English learning even during their college years (Wang and Jiang, 2021; Xiao, 2021), mainly due to serious English learning barriers caused by the English learning environment and exam mechanisms (Yang and Chen, 2016). The main concerns in academic area regarding English learning anxiety include: (1) the causes of anxiety. An in-depth exploration was conducted on the sources and influencing factors of English learning anxiety (Huang, 2021; AL-Qadri et al., 2023). These factors include learners’ personal characteristics (such as personality, learning foundation, learning motivation, etc.), external environment (such as teachers’ teaching methods, learning resources, etc.), and learning tasks (such as task difficulty, time constraints, etc.). (2) The impact of anxiety on English learning. High anxiety is believed to hinder the learning process and interfere with students’ reception and acquisition of language materials (Yu et al., 2015). In contrast, low anxiety is considered beneficial for second language acquisition (Yu, 2022). (3) Identification and assessment of anxiety. Identifying and evaluating English learning anxiety has also received attention from researchers (Zhang, 2008). This involves assessing students’ anxiety levels through observing their behavior and asking about their feelings, in order to provide them with timely help and intervention. (4) Strategies to reduce English learning anxiety. Some studies have found that teachers can reduce students’ English learning anxiety by using certain teaching strategies (Chen et al., 2022). For example, creating a safe and positive learning environment, providing support and encouragement, designing interesting and challenging learning tasks, and providing timely and positive feedback. In recent years, second language acquisition has shifted toward studying positive psychology (Shao et al., 2020; Mac Intyre, 2021), focusing on emotions, engagement, and teacher-student relationships, and emphasizing the role of emotions in language learning (Li and Xu, 2019; Fang and Tang, 2021). Therefore, it is of great significance to clarify the impact mechanism of teacher care behavior on English learning anxiety.

## Literature

### EFL learning anxiety

Anxiety is a psychological concept that refers to the fear and unease emotions that arise from an individual’s inability to evade behavior and cope with expectations or premonitions of setbacks. Anxiety is an important emotional factor that affects students’ learning, which can seriously affect academic performance (Tang and He, 2023). English learning anxiety is a specific emotional reflection originating from the English classroom environment, characterized by nervousness, anxiety, worry and fear, including communication anxiety, exam anxiety, and negative evaluation fear (Horwitz et al., 1986). Students are prone to anxiety in EFL learning (Aichhorn and Puck, 2017), and low proficiency foreign language learners are more likely to develop anxiety tendencies (Su, 2022). For a long time, English learning anxiety has been a focus of attention in the academic area (Horwitz et al., 1986; Dewaele and Mac Intyre, 2014; Yu et al., 2015; He, 2022; Gordani and Sadeghzadeh, 2023; Yu et al., 2023). It is believed that the causes of English learning anxiety include language cognitive impairment, insufficient expression and communication skills, weak competitive awareness, low self-efficacy, and dull teaching methods, etc. (Horwitz et al., 1986).

Research has found that English learning anxiety has seriously affected students’ English learning (Li and Liu, 2013; Mohammed et al., 2021), with some students developing a form of learning helplessness and frequent communication barriers in English (Ma et al., 2009; Tan and Xie, 2020). Moreover, long-term English learning anxiety can lead to a decrease in self-efficacy (Li and Liu, 2013), learning burnout (Jing, 2021), and affect academic performance (Xu, 2023; Zhang, 2023) and learning engagement (Dong and Zhang, 2023). However, some qualitative studies have also found that moderate EFL learning anxiety can facilitate foreign language learning (Yu, 2022).

### The mediating effect of teacher care behavior

Teacher care behavior originates from the feelings, thoughts, and activities of teachers that motivate or help learners with all their desires (O’Connor, 2008). Teacher care behavior is the behavior taken by teachers to establish a good relationship with students and ensure that expectations will occur (Lei et al., 2015). Essentially, teacher care refers to the emotions and attitudes that teachers hold toward students’ learning. During the teaching process, teachers show concern, understanding, respect, support, and encouragement to students through verbal and nonverbal behaviors. Teacher care behavior is conducive to the establishment of good teacher-student relationship. Through active interaction with teachers, students can achieve emotional and attitudinal stability, stimulate effective learning behavior (Pekrun and Schutz, 2007; Trigueros et al., 2020), and promote active student participation (Huang and Meng, 2017). Self-determination theory believes that teacher care behavior is an important driving factor for developing students’ intrinsic motivation, which can stimulate students’ learning related abilities and self-confidence, and enhance their self-efficacy (Havik and Westergård, 2020).

Studies have found that teacher care behavior has a positive impact on academic performance (Lei et al., 2015), which can promote students’ psychological capital to increase learning investment (Huang and Meng, 2017), enhance self-efficacy (Ye et al., 2017), alleviate learning pressure (Fu and Zhang, 2023; Jiang et al., 2023), and promote students’ mental health (Zheng et al., 2017; Kelly et al., 2022). Previous studies have shown that in EFL learning, teachers’ emotional support plays a moderating role in students’ self-efficacy and online English learning burnout (Yang et al., 2022), and teacher care behavior can promote students to increase their engagement in English learning (Sun, 2021).

### The mediating effect of learning engagement

Learning engagement is the degree of participation of students in educational tasks and activities (Heflin et al., 2017). It is an important potential variable for students to form learning motivation and optimize learning outcomes (Reschly and Christenson, 2012), as well as an important observational indicator during the learning process, and a key factor in students’ achieving academic success (Liu, 2023). Schaufeli et al. (2002) believes that learning engagement includes three dimensions: vigor, dedication, and absorption. Hart et al. (2011) measured students’ learning engagement from three aspects: emotion, cognition, and behavior. Lam et al. (2013) believes that the higher the motivational characteristics, the higher the level of engagement, and the closer the relationship between students, teachers, and peers, the higher the emotional and cognitive engagement. The three are interdependent. Behavioral engagement is the carrier of cognitive and emotional engagement and emotional engagement influences behavioral engagement through cognitive engagement. The degree of learning engagement has a direct impact on learning outcomes (Chi and Wylie, 2014), and learning engagement affects students’ self-learning regulation (An et al., 2023).

Previous studies have found that English learning engagement positively predicts learning satisfaction, and both emotional and cognitive engagement have a positive impact on behavioral engagement. Cognitive engagement is the mediator between emotional engagement and behavioral engagement (Ren, 2023). There is a positive correlation between learning engagement and learning strategies (Abubakar, 2017), which jointly predicts learning performance (Alqarni, 2023). Learning behavior engagement directly determines the level of learning engagement and affects the use of learning strategies (Abulela et al., 2023). For example, emotional engagement affects the use of emotional strategies, while cognitive engagement affects the use of memory strategies.

### The mediating effect of learning strategies

Language learning strategies are very important influencing factors in language learning, which are specific learning actions that language learners intentionally take to promote the acquisition, understanding, storage, and retrieval of information (Weinstein and Mayer, 1986). According to Oxford’s (1990) classification, language learning strategies include memory strategies, cognitive strategies, compensation strategies, metacognitive strategies, affective strategies, and communicative strategies. Research has found that learners’ use of language learning strategies is influenced by various factors, such as emotional factors (anxiety, confidence) (Krashen, 2009), learning motivation (Oxford, 1990), learning habits (Jiang, 2006), and language proficiency (Peng, 2023). Among them, learning anxiety is one of the most important factors affecting the use of language learning strategies (Nishitani and Matsuda, 2011; Xiong, 2011). Experiments have shown that anxiety is accompanied by a decrease in learning and memory abilities (Lu et al., 2022). In contrast, memory capacity has a certain regulatory effect on anxiety (Hu and Zhou, 2022). Learning strategies not only affect academic performance (Abimbade, 2023; Alqarni, 2023), but also predict learning engagement (Abulela et al., 2023), such as cognitive strategies affecting students’ learning engagement (Kim and Kim, 2016). Good learning strategies can help students reduce learning anxiety (Geng, 2021; Ji, 2022).

Based on the above literature review, it can be seen that existing research only preliminarily discussed the influence of teacher care on English learning engagement, with little exploration of the mechanism of teacher care’s impact on EFL learning anxiety, and little discussion on the relationship between teacher care behavior and learning engagement, learning strategies, and EFL learning anxiety.

Thus, this paper proposes the following assumptions:

*H1*: There is a negative correlation between teacher care behavior and EFL learning anxiety.

*H2*: Learning engagement plays a mediating role in teacher care behavior and EFL learning anxiety.

*H3*: Learning strategies play a mediating role in teacher care behavior and EFL learning anxiety.

*H4*: Teacher care behavior plays a chain mediating role in EFL learning anxiety through learning engagement and learning strategies.

## Research design

### Research sample

This study uses a mediating effect model to explore the impact and mechanism of teacher care behavior on EFL learning anxiety. The mediating variables are learning engagement and English learning strategies. The independent variable is teacher care behavior, and the dependent variable is EFL learning anxiety. Subsequently, a convenient cluster sampling method was used to select 522 college students (*M* = 20.06 years old, SD = 1.21) from three universities in the capital cities of the central and southeastern provinces as survey subjects. They filled out the Teacher Care Behavior Scale, Foreign Language Classroom Anxiety Scale, Utrentwork Engagement Scale-Student, and Strategy Inventory for Language Learning Scale online, respectively. A total of 504 valid samples were obtained in this survey (excluding samples with online answering time less than 180 s), with an effective rate of 96.55%. Among them, there are 263 female students (52.18%) and 241 male students (47.82%); 138 English majors (27.38%) and 366 non-English majors (72.62%).

## Measures

### Teacher care behavior scale

Teacher care behavior was assessed with Lei’s (2014) “Teacher Care Behavior Scale” (Chinese version) which consists of 18 items and three dimensions: conscientiousness, support, and inclusiveness. Participants were asked to report their English teacher’s behavior in the past year (e.g., the teacher takes time to know me). The scores were rated using Likert 5-point scale, from “1” strongly disagree to “5” strongly agree, the higher the score, the higher the teacher’s caring behavior. The Cronbach’s alpha of this scale is 0.965, and the Cronbach’s alpha coefficients for each dimension are 0.912, 0.944, and 0.941, respectively. This study conducted exploratory and confirmatory factor analysis on the scale, and the results were as follows: KMO was 0.957, the spherical degree of Bartlett test for sig value.000, at 0.05 significant level; *χ^2^/df* = 2.746, GFI = 0.912, CFI = 0.931, SRMR = 0.024, RMSEA = 0.057.

### Foreign language classroom anxiety scale

Students’ EFL learning anxiety was assessed with Horwitz et al.’s (1986) “Foreign Language Classroom Anxiety Scale-FLCAS” [Chinese Version by Wang (2003)], which consists of 33 items and four dimensions: worry, nervousness, fear of speaking English, and fear of classroom questioning. Students were asked to report their EFL learning anxiety in the past year (e.g., I have no confidence when speaking English in foreign language classroom). The scores were rated using Likert 5-point scoring system, from “1” being very inconsistent to “5” being very consistent. The higher the score, the more anxious foreign language learning anxiety is. The Cronbach’s alpha of this scale is 0.963, and the Cronbach’s alpha coefficients for each dimension are 0.903, 0.908, 9.02, and 0.905, respectively. This study conducted exploratory and confirmatory factor analysis on the scale, and the results were as follows: KMO was 0.971, the spherical degree of Bartlett test for sig value.000, at 0.05 significant level; *χ^2^/df =* 2.027, GFI = 0.918, CFI = 0.945, SRMR = 0.028, RMSEA = 0.036.

### Utrentwork engagement scale-student

Students’ learning engagement was assessed with Schaufeli’s “Utrechtwork Engagement Scale-Student-UWES-S” [Chinese Version by Li and Huang (2010)], which consists of 17 items and three dimensions: vigor, dedication, and absorption. Students were asked to report their learning engagement in the past year (e.g., I feel full of energy when learning English). The scores were rated using Likert 7-point scoring, from “1″ the situation never occurs to “7″ the situation always occurs, with higher scores indicating greater learning engagement. The Cronbach’s alpha of this scale is 0.961, and the Cronbach’s alpha coefficients for each dimension are 0.938, 0.922, and 9.01, respectively. This study conducted exploratory and confirmatory factor analysis on the scale, and the results were as follows: KMO was 0.953, the spherical degree of Bartlett test for sig value.000, at 0.05 significant level; *χ^2^/df =* 2.892, GFI = 0.912, CFI = 0.937, SRMR = 0.039, RMSEA = 0.063.

### Strategy inventory for language learning (SILL) scale

Students English learning strategy was assessed with Oxford’s (1990) “Strategy Inventory for Language Learning Scale” (Chinese Version), which consists of 50 items and six dimensions: memory, cognition, compensation, metacognition, emotion, and communication. Students were asked to report their English learning strategy in the past year (e.g., In order to better memorize the words, I write down the new words on the card). The scores were rated using Likert’s 5-point scoring system, from “1” very inconsistent to “5” very consistent. The higher the score, the more learning strategies there are. The Cronbach’s alpha of this scale is 0.974, and the Cronbach’s alpha coefficients for each dimension are 0.929, 0.917, 0.898, 0.920, 0.907, and 0.908, respectively. This study conducted exploratory and confirmatory factor analysis on the scale, and the results were as follows: KMO was 0.966, the spherical degree of Bartlett test for sig value.000, at 0.05 significant level; *χ^2^/df =* 2.137, GFI = 0.925, CFI = 0.953, SRMR = 0.025, RMSEA = 0.049.

### Research procedures and statistical analysis

Informed consent was obtained from each participant before completing the online questionnaires, which required approximately 15 min. The valid data was obtained for common method bias testing. Factor analysis was conducted using Harman’s single factor analysis on the Teacher Care Behavior Scale, FLCAS, UWES-S, and SILL Scale. The results showed that a total of 16 common factors with eigenvalues greater than 1 were generated, of which the variance explained by the first common factor after rotation was 18.11%, much less than the 40% critical standard (Tang and Wen, 2020). Therefore, it can be inferred that there is no significant common method bias in this study. First, the inter subject effects were examined through demographic and professional variable differences statistics. Second, descriptive statistics were conducted on each variable to test their correlation. Finally, chain mediated effect analysis and testing were conducted using the SPSS 23.0 software Process plugin, and Model 6 was selected and the 95% confidence interval of parameter estimation was obtained using the 5,000 bootstrap method of repeated sampling.

## Results

### Intersubjective effect test

By conducting statistical analysis of the differences between demographic and professional variables (see Table 1), the author examined whether the main effects of gender and major variables were significant, that is, conducting inter subject effects tests on four variables: gender, major and teacher care behavior, foreign language learning anxiety, learning engagement, and English learning strategies. (1) Compared with boys, the gender main effects of female students in EFL learning anxiety were significant (*F* = 4.548, *p* < 0.05), The main effects of the other three variables were not significant; (2) Compared with English majors, non-English majors had a significant main effect on EFL learning anxiety (*F* = 4.823, *p* < 0.05), while the other three variables had no significant main effect.

**Table 1:** Intersubjective effect test of gender and major (M ± SD).

		Care	Anxiety	Engagement	Strategy
gender	*F* (*n* = 263)	75.825 ± 13.191	65.076 ± 21.499	75.521 ± 17.645	165.825 ± 32.697
	*M* (*n* = 241)	75.905 ± 11.672	62.639 ± 19.031	77.253 ± 20.156	168.709 ± 33.734
major	EM (*n* = 138)	75.703 ± 12.572	61.304 ± 23.319	76.319 ± 20.035	167.326 ± 42.905
	NE (*n* = 366)	75.924 ± 12.456	64.893 ± 19.087	76.361 ± 18.466	167.159 ± 28.768
F_(g)_		0.153	4.548^*^	0.635	0.552
F_(m)_		0.002	4.823^*^	0.004	0.016

*p < 0.05. Care, teacher care behavior; Anxiety, EFL learning anxiety; Engagement, learning Engagement; Strategy, English learning strategy; g, gender; m, major; F, female; M, male; EM, English major; NE, Non-English; Gender and major were dummy coded such that 1 = Female, 2 = Male, 3 = English major, 4 = non-English major.

### Correlation analysis between variables

Control variables such as gender and major, and analyze the correlation between the main variables using partial correlation analysis. From Table 2, it can be seen that teacher care behavior, learning engagement, and English learning strategies are significantly positively correlated with each other (*r* = 0.332 ~ 0.588, *p* < 0.01), and negatively correlated with EFL learning anxiety (*r* = −0.328 ~ −0.256, *p* < 0.01). Among them, there is a significant negative correlation between teacher care behavior and EFL learning anxiety (*r* = −0.309, *p* < 0.01), indicating that theoretical hypothesis H1 has been validated.

**Table 2:** Correlation analysis of TCB, EFLLA, LE, and ELS.

		*M*	SD	1	2	3	4
1	Care	75.863	12.476	1			
2	Anxiety	63.911	20.373	−0.309^**^	1		
3	engagement	76.349	18.888	0.332^**^	−0.256^**^	1	
4	Strategy	167.204	33.195	0.359^**^	−0.328^**^	0.588^**^	1

**p < 0.01. TBC, teacher care behavior; EFLLA, EFL learning anxiety; LE, learning engagement; ELS, English learning strategy.

From Table 3, it can be seen that teacher care behavior which includes three dimensions: conscientiousness, support, and inclusiveness, learning engagement which includes three dimensions: vigor, dedication, and absorption, and English learning strategies which include six dimensions: memory, cognition, compensation, metacognition, emotion, and communication, are significantly positively correlated with each other (*r* = 0.195 ~ 0.893, *p* < 0.01), while English learning anxiety which includes worry, nervousness, fear of speaking English and fear of classroom questioning are significantly negatively correlated with each other (*r* = −0.359 ~ −0.126, *p* < 0.01), further confirming the theoretical hypothesis H1.

**Table 3:** Correlation analysis of CS, SU, IN, WO, ON, VI, DE, and AB.

		1	2	3	4	5	6	7	8	9	10	11	12	13	14	15	16
1	CS	1															
2	SU	0.771^**^	1														
3	IN	0.813^**^	0.743^**^	1													
4	WO	−0.321^**^	−0.271^**^	−0.359^**^	1												
5	ON	−0.276^**^	−0.240^**^	−0.287^**^	0.822^**^	1											
6	AS	−0.177^**^	−0.175^**^	−0.210^**^	0.621^**^	0.720^**^	1										
7	AA	−0.204^**^	−0.203^**^	−0.239^**^	0.721^**^	0.781^**^	0.826^**^	1									
8	VI	0.357^**^	0.310^**^	0.340^**^	−0.254^**^	−0.256^**^	−0.195^**^	−0.224^**^	1								
9	DE	0.244^**^	0.236^**^	0.250^**^	−0.186^**^	−0.178^**^	−0.126^**^	−0.172^**^	0.794^**^	1							
10	AB	0.272^**^	0.257^**^	0.270^**^	−0.236^**^	−0.229^**^	−0.180^**^	−0.233^**^	0.720^**^	0.798^**^	1						
11	ME	0.323^**^	0.289^**^	0.325^**^	−0.325^**^	−0.340^**^	−0.273^**^	−0.328^**^	0.565^**^	0.461^**^	0.508^**^	1					
12	CG	0.286^**^	0.269^**^	0.282^**^	−0.268^**^	−0.267^**^	−0.185^**^	−0.235^**^	0.530^**^	0.404^**^	0.440^**^	0.809^**^	1				
13	CM	0.217^**^	0.195^**^	0.201^**^	−0.205^**^	−0.199^**^	−0.166^**^	−0.212^**^	0.336^**^	0.226^**^	0.215^**^	0.494^**^	0.543^**^	1			
14	MT	0.290^**^	0.299^**^	0.294^**^	−0.246^**^	−0.258^**^	−0.209^**^	−0.248^**^	0.558^**^	0.440^**^	0.466^**^	0.746^**^	0.715^**^	0.576^**^	1		
15	EM	0.323^**^	0.318^**^	0.313^**^	−0.294^**^	−0.284^**^	−0.225^**^	−0.276^**^	0.577^**^	0.461^**^	0.529^**^	0.825^**^	0.766^**^	0.508^**^	0.804^**^	1	
16	CO	0.289^**^	0.282^**^	0.296^**^	−0.299^**^	−0.279^**^	−0.225^**^	−0.270^**^	0.573^**^	0.450^**^	0.502^**^	0.816^**^	0.757^**^	0.472^**^	0.789^**^	0.893^**^	1
	Ske	−0.848	−0.688	−0.816	0.205	0.134	0.066	0.056	0.040	−0.121	0.045	0.149	0.131	0.266	0.192	0.207	0.057
	Kur	0.696	−0.021	0.241	−1.010	−1.243	−1.456	−1.265	0.330	0.612	0.343	0.678	0.852	0.468	0.914	0.651	0.566
	*M*	29.706	24.964	21.193	22.403	17.653	11.833	12.022	27.316	22.228	26.806	29.960	46.784	21.069	30.232	19.544	19.615
	SD	4.882	4.819	3.821	7.595	6.197	4.569	4.220	7.131	6.275	7.132	7.168	9.852	4.121	6.693	4.968	4.984

CS, Conscientiousness; SU, Support; IN, inclusiveness; WO, worry; ON, on edge; AS, Afraid of speaking English; AA, Afraid of asking questions; VI, vigor; DE, dedication; AB, absorption; ME, memory; CG, cognition; CM, compensation; MT, metacognition; EM, emotion; CO, communication; Ske, Skewness; Kur, Kurtosis.

### Analysis of chain mediation model

From Table 4, it can be seen that in Equation 1, teacher care significantly positively predicts learning engagement (*β* = 0.109, *t* = 3.296, *p* < 0.001). Equation 2 shows that teacher care behavior is significantly positively predictive of English learning strategies (*β* = 0.073, *t* = 3.810, *p* < 0.001), while learning engagement significantly positively predicts English learning strategies (*β* = 0.943, *t* = 14.769, *p* < 0.001). In Equation 3, when teacher care behavior, learning engagement, and English learning strategies are all included in the regression equation, teacher care behavior (*β* = −0.124, *t* = −4.735, *p* < 0.001), learning engagement (*β* = −0.237, *t* = −5.918, *p* < 0.001), English learning strategies (*β* = −0.307, *t* = −5.200, *p* < 0.001), all three significantly negatively predict EFL learning anxiety.

**Table 4:** A chain mediated model of TCB and EFLLA.

	Equation 1: LE			Equation 2: ELS			Equation 3:EFLLA		
	*β*	SE	*t*	*β*	SE	*t*	*β*	SE	*t*
Constant	−9.061	7.135	−1.263	30.276	10.213	2.964	−6.406	4.448	−1.440
TCB	0.109	0.033	3.296^***^	0.073	0.019	3.810^***^	−0.124	0.026	−4.735^***^
LE				0.943	0.063	14.769^***^	−0.237	0.040	−5.918^***^
ELS							−0.307	0.059	−5.200^***^
Gender	−0.492	2.017	−0.244^***^	5.718	2.882	1.984	−0.755	1.249	−0.604
Major	2.629	2.259	1.163	−10.395	3.233	−3.214	2.099	1.410	1.488
*R* ^2^	0.068			0.385			0.189		
*F*	12.123^***^			78.043^***^			23.314^***^		

***p < 0.001. TBC, teacher care behavior; EFLLA, EFL learning anxiety; LE, learning engagement; ELS, English learning strategy.

**Figure 1:**
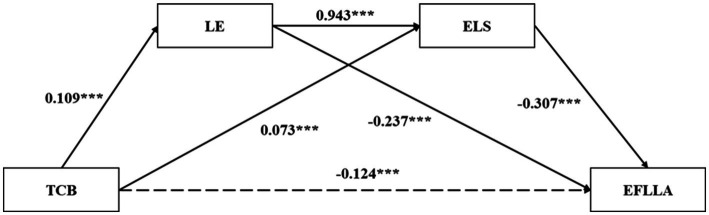
A chain mediated model of TCB and EFLLA.

According to Table 5 and Figure 1, it can be seen that learning engagement and English learning strategies play a mediating role between teacher care behavior and EFL learning anxiety, with a total indirect mediating effect value of −0.065, accounting for 34.39% of the total effect −0.189. At this point, the mediating effect consists of three pathways: (1) learning engagement serves as a mediator, and the indirect effect generated by teacher care behavior → learning engagement → EFL learning anxiety is −0.026 (−0.047, −0.009) and the effect amount is 13.76%, indicating that the theoretical hypothesis H2 has been verified; (2) The indirect effects of teacher care behavior, English learning strategy, and EFL learning anxiety as mediators are −0.023 (−0.041, −0.008) and the effect amount is 12.17%, indicating that the theoretical hypothesis H3 has been validated; (3) The indirect effects of teacher care behavior, learning engagement, English learning strategies, and EFL learning anxiety are −0.016 (−0.028, −0.006) and the effect amount is 8.46%, respectively, and the theoretical hypothesis H4 is verified. Meanwhile, the Bootstrap 95% CI of the mediating effect did not include 0 and reached a significant level.

**Table 5:** The chain mediation effect test of TCB and EFLLA.

Effect	Path	EV	ES	95%CI	95%CI
				Lower	Upper
Direct	TCB → EFLLA	−0.124	65.61%	−0.176	−0.073
Indirect	TCB → LE → EFLLA	−0.026	13.76%	−0.047	−0.009
	TCB → ELS → EFLLA	−0.023	12.17%	−0.041	−0.008
	TCB → LE → ELS → EFLLA	−0.016	8.46%	−0.028	−0.006
Total indirect		−0.065	34.39%	−0.092	−0.041
Total		−0.189		−0.240	−0.138

TCB, teacher care behavior; EFLLA, EFL learning anxiety; EV, estimated value; ES, effect size; LE, learning engagement; ELS, English learning strategy.

## Discussion

### The relationship between teacher care behavior and EFL learning anxiety

The inter subject effect test of this study shows that there is a significant gender and major effect between female students and non-English majors in EFL learning anxiety. This result indicates that female students and non-English major students are more likely to experience learning anxiety in the EFL classroom, which also confirms the correlation between anxiety and gender (Bensalem, 2018). The relevant analysis of this study shows that teacher care behavior, learning engagement, and English learning strategies are significantly positively correlated with each other, while they are significantly negatively correlated with EFL learning anxiety. Among them, there is a significant negative correlation between conscientiousness, support, inclusiveness, vigor, dedication, absorption, memory, cognition, compensation, metacognition, emotion, communication and worry, nervousness, fear of speaking English, fear of classroom questioning. This deeply indicates that the first three have a close positive correlation and a negative correlation with EFL learning anxiety, which also provides data support for testing the chain mediated effect. Previous studies have shown that the positive event context in which an individual is exposed can affect their state of mind, leading to changes in their behavior (Rosenhan et al., 1980). Foreign language learning anxiety is a negative state of mind generated by subtle cognitive processing efforts of learners in the process of processing input and output information (Sparks and Ganschow, 1991). It is worry and fear caused by individuals’ negative premonition of results (Tao and He, 2021), and a specific emotion generated during foreign language classroom learning (Dong, 2021). In fact, the caring behavior of teachers in the context of foreign language learning is a concrete manifestation of a positive state of mind which can influence the learners to a large extent. The results of this study confirm the negative correlation between teacher care behavior and EFL learning anxiety, which proved the positive effect of teacher care behavior in EFL learning context.

### The mediating effect of learning engagement and English learning strategies

The results of this study indicate that learning engagement and English learning strategies partially mediate the relationship between teacher care behavior and EFL learning anxiety, respectively. That is to say, teacher care behavior can directly affect EFL learning anxiety, or indirectly affect EFL learning anxiety through influencing learning engagement and English learning strategies. On the one hand, the stronger a learner’s vigor, absorption, and internal mobilization, the more sustainable, explicit, positive, and stable their language learning tendencies are, thereby offsetting some negative emotions such as anxiety and burnout (Bailey et al., 1998). On the other hand, learners who possess good cognitive information processing strategies and sufficient memory carrying capacity in the process of foreign language reading and oral communication (Dewaele and Mac Intyre, 2014) can effectively reduce the probability of foreign language learner’s learning anxiety (Dewaele et al., 2019). In foreign language classroom teaching, it is possible to reduce concerns, nervousness, and fear of questioning in foreign language learning by enhancing learners’ enthusiasm and focus, as well as improving their cognitive and memory methods. The research conclusion shows that the characteristics of teacher care behavior in foreign language classroom teaching, such as encouragement, tolerance, and responsibility, essentially belong to the category of positive mood, and can also affect learning engagement and learning strategies, thereby reducing the level of foreign language learner’s learning anxiety.

### The chain mediating effect of learning engagement and English learning strategies

This study found that teacher care behavior can affect EFL learning anxiety through the chain mediating effect of learning engagement and English learning strategies. Specifically, as the teacher’s sense of responsibility, inclusivity, and support become apparent in classroom teaching, this positive emotion will encourage learners to overcome anxiety, encourage themselves, enhance their confidence, arrange learning in a planned manner, establish learning priorities, and strive to overcome their own shortcomings, etc. That is to say, teacher care behavior is not only a resource for foreign language classroom teaching environment, but also a situational variable that has a significant positive effect on the cognitive to behavioral process. Here, a general learning model can be used to explain (Buckley and Anderson, 2006), that teachers adjust their internal psychological activities and act on learners’ psychological processes, which is conducive to the generation of positive psychology (PP) for learners. It is why PP has become more and more popular in second and foreign language teaching and acquisition recent years, because PP can generate positive language learning activities (Mac Intyre and Mercer, 2014; Dewaele et al., 2019). That is, language learners invest a large amount of time to promote the improvement of English learning strategies and improve their self-confidence, thereby reducing anxiety such as worry or fear of questioning in the classroom with the changing of their mood to positive psychology. This is not only a cognitive processing course, but also a process of mind improvement. Although EFL learners often experience negative emotions due to questioning in foreign language classrooms, under the influence of PP, learners actively enhance their level of focus or vigor in learning, actively participate in learning activities, form initiative from different levels of cognition, emotion, and behavior, promote language learning, and enhance cognitive processing and memory abilities in foreign language learning (Philp and Duchesne, 2016; Ellis, 2019). In summary, teachers are the key factors affecting the effectiveness of classroom teaching (Huang and Xin, 2007). Through their own behavior support and encouragement, teachers encourage learners to invest as much energy and strategies as possible, thereby reducing the level of EFL learning anxiety.

This study examined the chain mediating effect of learning engagement and English learning strategies on teacher care behavior and EFL learning anxiety, and found that there are the following pathways to the impact of teacher care behavior on EFL learning anxiety: encouraging, accommodating, and other behaviors can enable learners to invest more energy, focus, and other psychological capital, continuously improving individual cognitive processing and memory abilities, thereby reducing concerns in foreign language classrooms anxious emotions such as fear of asking questions. This pathway highlights the co-mediating effect of learning engagement and learning strategies, not only revealing the interrelationship between teacher care behavior and foreign language learner’s learning anxiety, but also providing a basis for foreign language classroom learning theory.

## Conclusion

Firstly, this study explores the chain mediating role of learning engagement and English learning strategies in teacher care behavior and EFL learning anxiety, which not only enriches previous research but also reveals the impact mechanism of teacher care behavior on EFL learning anxiety. Previous studies have focused on the relationship between teacher care behavior and learning engagement, as well as English learning strategies, with less emphasis on incorporating EFL learning anxiety into the research scope. In essence, in foreign language classroom teaching, learners are always in a passive state to participate in classroom interaction, leading to frequent burnout and anxiety in foreign language learning. Therefore, this study aims to discuss the phenomenon of EFL learning anxiety and supplement previous studies.

Secondly, this study conducted inter subject effects testing, variable correlation analysis, and mediation effects testing, and the results all supported the proposed theoretical hypothesis. From this, the following conclusion can be drawn: teacher care behavior is considered as a category of psychological capital in EFL classrooms, and is a key element in reducing EFL learning anxiety levels. Teacher care behavior enhances learners’ learning engagement and improves their English learning strategies, thereby reducing EFL learning anxiety levels. Although classroom psychological capital, individual energy, and cognitive processing ability have independent mechanisms in reducing EFL learning anxiety levels, this study verified through chain mediation that their combined effects are more significant.

This study further indicates that teacher classroom teaching design should fully consider intervention measures for EFL learning anxiety, facilitate the application of corresponding incentive measures to learners, enhance their confidence and self-efficacy, and thereby improve individual cognitive processing and memory strategies.

### Implications

The empirical data of this study indicates that teacher care behavior has a significant negative predictive effect on English learning anxiety. Teacher care behavior can reduce students’ EFL learning anxiety through joint mediating effect of learning engagement and English learning strategies. This indicates that teacher caring behavior can enhance students’ positive emotional experience and their learning motivation, increase their learning engagement, improve individual cognitive processing and memory abilities, change learning methods and strategies, and alleviate negative emotions such as worry and fear of questioning in foreign language classrooms. Therefore, this study provides a basis for foreign language classroom teaching and learning theory. Given all of this, this study proposes two suggestions for optimizing foreign language teaching and learning.

First, students’ English learning anxiety can be reduced due to the caring behavior of teachers. Therefore, teachers should increase emotional support, give more attention and understanding of students’ learning situation, learning status and level of anxiety, and provide learning suggestions and guidance to help them alleviate their EFL learning anxiety. In class, teachers try to create a relaxed and pleasant learning atmosphere for students. For those who are prone to nervousness and fear of asking questions, teachers can encourage them to express themselves bravely, such as speaking in groups, reciting texts, giving rewards for their participation, etc., to improve students’ confidence and sense of achievement in English learning and improve their English learning ability.

Due to the joint mediation of learning engagement and English learning strategies in reducing students’ EFL learning anxiety, teachers can help students improve their learning engagement, encourage them to invest more energy and focus in English learning, such as formulating detailed English learning plans, preparing the study in advance, identifying learning difficulties, etc., and encourage them to think more in English and increase their output rate. Teachers can also give them English learning strategies training, help students change their English learning methods, improve their English learning efficiency, and become a successful language learner.

### Limitations

This study used a scale scoring test to examine the chain mediating effect of learning engagement and English learning strategies on teacher care behavior and EFL learning anxiety. EEG indicators were not included in the scope of the test. Therefore, it is necessary to use more physiological indicators as mediating variables in future research.

## Data availability statement

The raw data supporting the conclusions of this article will be made available by the authors, without undue reservation.

## Ethics statement

Ethical review and approval were not required for the study on human participants in accordance with the local legislation and institutional requirements. The participants provided their online informed consent to participate in this study.

## Author contributions

DW: Writing – original draft, Writing – review & editing.
